# Monitoring Systemic Inflammatory Indices and Retinal Changes Over Time Following Initial Diagnosis of Diabetic Retinopathy

**DOI:** 10.1155/joph/5542064

**Published:** 2026-04-16

**Authors:** Emre Aydın, Şaban Kılıç, Çiğdem Deniz Genç

**Affiliations:** ^1^ Department of Ophthalmology, Samsun Education and Research Hospital, Samsun, Turkey; ^2^ Department of Ophthalmology, Samsun Training and Research Hospital, Samsun, Turkey, samsundh.gov.tr

**Keywords:** diabetic retinopathy, NLR, optical coherence tomography, SII, systemic inflammation

## Abstract

**Objective:**

This study aimed to evaluate changes in systemic inflammatory indices and retinal structural parameters in newly diagnosed diabetic retinopathy (DR) patients before and after intravitreal anti‐VEGF treatment.

**Material and Methods:**

A total of 308 participants (157 DR patients and 151 healthy controls) were enrolled. Systemic inflammatory status was assessed using hematologic indices derived from routine complete blood count parameters. Optical coherence tomography (OCT) was used to measure macular thickness. DR patients were evaluated at three time points: before treatment, 1 month, and 3 months after anti‐VEGF injection.

**Results:**

Baseline inflammatory indices (NLR, MLR, PLR, and SII) and macular thickness were significantly higher in the DR group compared to controls (*p* < 0.001). Following treatment, a progressive decrease in both inflammatory indices and macular thickness was observed. Pretreatment NLR and SII values showed high diagnostic performance in differentiating DR (AUC: 0.925 and 0.838, respectively). Multivariate analysis revealed that NLR, MLR, and HbA1c were independent predictors of macular thickness.

**Conclusion:**

Systemic inflammatory activity is closely associated with both the onset and progression of DR. The evaluation of inflammatory indices such as NLR and SII together with OCT findings may support diagnosis and treatment follow‐up in clinical practice.

## 1. Introduction

As a frequent microvascular sequela of diabetes mellitus (DM), diabetic retinopathy (DR) remains a major global cause of vision loss, particularly among individuals of working age [1]. Damage to the retinal vasculature gradually leads to structural and functional deterioration, negatively affecting visual quality [2]. This process is closely associated with both the duration and control level of diabetes. Although it is often asymptomatic in the early stages, it may result in significant vision loss in more advanced stages [3].

Beyond vascular changes, systemic inflammatory processes are thought to contribute to the development of DR [4]. Diabetes is considered a chronic inflammatory condition, which may increase retinal vascular permeability and accelerate the development of retinopathy [5]. Systemic inflammatory status can be evaluated through markers obtained from peripheral blood analyses. Specifically, hematologic markers are calculated from routine complete blood count parameters and are extensively used to assess the degree of systemic inflammation [6, 7].

Optical coherence tomography (OCT) plays a crucial role in monitoring intraocular structural changes. Findings such as retinal thickness measurements and fluid accumulation in the macular region are critical for assessing treatment response [8]. In patients with DR, both systemic inflammatory status and retinal structure may change over time. Assessing these parameters together may improve insight into disease progression.

Recent evidence suggests that the interaction between systemic inflammatory burden and retinal microarchitecture is dynamic rather than static, particularly in the early phases of DR [9]. Fluctuations in circulating inflammatory cell profiles may reflect subclinical endothelial dysfunction and low‐grade immune activation, which can precede overt microvascular damage [10]. These systemic alterations may influence retinal homeostasis through inflammatory mediators, oxidative stress pathways, and disruption of the blood–retinal barrier. Therefore, longitudinal evaluation of inflammatory indices in parallel with quantitative retinal imaging parameters may provide complementary information beyond cross‐sectional assessments, enabling a more refined understanding of early disease activity and treatment‐related biological responses.

This investigation aimed to examine short‐term changes in hematologic inflammatory indices and OCT‐derived findings in individuals with newly diagnosed DR and evaluated prior to treatment and during follow‐up at 1 and 3 months. The results may help clarify early inflammatory activity and associated retinal structural alterations in DR.

## 2. Materials and Methods

### 2.1. Study Design and Ethical Approval

This study was designed as a single‐center, prospective cohort investigation. Data were collected at the Ophthalmology Clinic of Samsun Training and Research Hospital between November 1, 2024, and May 15, 2025. The protocol of this study received approval from the Samsun University Non‐Interventional Clinical Research Ethics Committee on June 5, 2024, under approval number GOKAEK 2024/11/5. Written informed consent was obtained from all participants.

### 2.2. Patient and Control Group Selection

The study population comprised patients with newly diagnosed DR. Inclusion criteria were diagnosis of type 2 DM, presence of DR confirmed by ophthalmologic examination, no prior anti‐VEGF therapy, and age ≥ 18 years. Exclusion criteria included coexisting retinal diseases (e.g., retinal vein occlusion), systemic inflammatory diseases (e.g., rheumatoid arthritis), and active infections. The control group was composed of healthy individuals with no history of systemic disease, infection, or inflammatory condition. Although no matching for age or sex was performed, the absence of diabetes, lack of systemic illness, and normal findings in ophthalmologic examination were the key criteria for control subjects. This allowed for a comparative analysis of inflammatory marker levels between the reference values of the control group and those of the patient group. Patients with DR were referred from the ophthalmology outpatient clinic to the internal medicine clinic for review of their treatment plans based on blood glucose monitoring results following eye examination and treatment.

### 2.3. Ophthalmological Evaluation

At baseline, all patients underwent fundus fluorescein angiography (FFA) for diagnostic confirmation and disease staging of DR. FFA was performed using a combined confocal scanning laser ophthalmoscopy and OCT system (Spectralis HRA + OCT; Heidelberg Engineering GmbH, Heidelberg, Germany). In the angiographic assessment, the arterial, venous, and late phases were evaluated in detail for leakage, ischemic areas, microaneurysms, intraretinal microvascular abnormalities (IRMA), venous beading, and signs of neovascular proliferation. At baseline FFA evaluation, no neovascularization of the disc (NVD) and no neovascularization elsewhere (NVE) were detected in any patient. The absence of angiographic neovascularization confirmed that none of the included patients met the criteria for proliferative DR, supporting the clinical and OCT‐based staging. The FFA findings were fully consistent with the fundoscopic examination, and no additional or subclinical proliferative changes were observed. The pivotal diagnostic role of fluorescein angiography in evaluating microvascular alterations in DR has also been emphasized in recent clinical guidelines [11]. Laser photocoagulation was performed only in cases where focal or panretinal intervention was deemed clinically necessary based on follow‐up examinations. All LP procedures were conducted after the initial anti‐VEGF injection, typically after the first‐month evaluation, when macular edema had regressed sufficiently to allow clear visualization of the retina. The grading of DR was based on a standardized clinical staging framework, in which disease severity was determined according to the International Clinical Diabetic Retinopathy Severity Scale, incorporating classification principles originally established by the Early Treatment Diabetic Retinopathy Study [12, 13]. Accordingly, Grade 1 (mild NPDR) was defined by the presence of microaneurysms only; Grade 2 (moderate NPDR) was defined by multiple microaneurysms, intraretinal hemorrhages, and mild‐to‐moderate IRMA; and Grade 3 (severe NPDR) was defined by features consistent with the ETDRS 4–2–1 rule, including extensive hemorrhages/MA, prominent venous beading, or marked IRMA. No patient demonstrated proliferative retinopathy at baseline or during staging.

### 2.4. Laboratory Analyses

Blood sampling was performed following a fasting period of approximately 8–12 h, with collections scheduled in the early daytime period for all study participants. Biochemical measurements were conducted on serum specimens using an automated chemistry analyzer (Cobas C702; Roche Diagnostics, Germany). The laboratory panel included glycated hemoglobin, fasting glucose, and lipid parameters encompassing total cholesterol as well as low‐ and high‐density lipoprotein fractions. Complete blood count analyses were carried out with an automated hematology system (BC‐6800; Mindray Bio‐Medical Electronics Co., China). Based on these hematological data, inflammatory indices—namely, the neutrophil‐to‐lymphocyte ratio, platelet‐to‐lymphocyte ratio (PLR), monocyte‐to‐lymphocyte ratio, and the systemic immune‐inflammation index (platelet × neutrophil/lymphocyte)—were calculated. Measurements were obtained at study entry and repeated at the first and third month in the patient cohort, whereas a single assessment was performed for control subjects, with all results documented through an electronic data recording system.

### 2.5. Statistical Analysis

All statistical computations were performed with the SPSS software package (IBM SPSS Statistics, Version 27.0; IBM Corp., Armonk, NY, USA). Prior to analysis, the normality of continuous variables was examined through the Shapiro–Wilk procedure. Descriptive statistics for normally distributed data were expressed as mean values with corresponding standard deviations, while categorical data were summarized as counts and proportions. Between‐group comparisons involving the control cohort and individuals with DR were conducted using parametric testing for independent samples. Temporal changes within the DR group were examined by means of repeated‐measures analysis of variance across baseline, first‐month, and third‐month assessments. In instances where the assumption of sphericity was not met, as determined by Mauchly’s test, adjusted degrees of freedom were applied using the Greenhouse–Geisser approach. To verify global time‐related effects, multivariate test statistics, including Wilks’ Lambda, were additionally reported. Patterns of longitudinal change were further characterized through polynomial contrast analyses. Associations between systemic inflammatory markers (NLR, MLR, PLR, and SII) and both glycemic control parameters and macular thickness measurements were explored using correlation analysis at baseline and at the 3‐month follow‐up. Diagnostic accuracy of pretreatment NLR and SII values for identifying DR was evaluated by receiver operating characteristic curve methodology, with area under the curve estimates, sensitivity, specificity, and optimal threshold values presented alongside 95% confidence intervals. Finally, multivariable linear modeling was employed to identify factors independently associated with baseline macular thickness, incorporating demographic, clinical, metabolic, and inflammatory variables. Statistical significance was defined as a two‐sided *p* value below 0.05. To evaluate longitudinal within‐group variation among participants with DR, analyses were planned across three predefined assessment occasions. Sample size adequacy was determined in advance through a statistical power estimation performed with G × Power software (Version 3.1; Heinrich Heine University Düsseldorf, Germany). The analytical framework was based on a within‐subjects repeated‐measures design. A medium magnitude effect (*f* = 0.25) was assumed, together with an intermeasurement correlation coefficient of 0.50 and an epsilon adjustment of 0.75 to account for potential violations of sphericity. To control the overall type I error rate across four primary hypotheses, the significance threshold was adjusted using a Bonferroni‐based approach, yielding an alpha level of 0.00125, while the desired power was set at 95%. Under these assumptions, the minimum number of participants required for adequate statistical sensitivity was estimated as 95 (critical *F* = 8.362; numerator df = 1.5; denominator df = 141; noncentrality parameter = 26.72; observed power = 0.951). The final DR cohort included 157 individuals, thereby exceeding the calculated sample size requirement.

## 3. Results

A total of 308 participants were included: 49.0% were in the control group, and 51.0% were in the DR group. The mean age of the study population was 61.6 ± 6.0 years, the mean BMI was 26.3 ± 4.4 kg/m^2^, and the mean duration of diabetes was 110.7 ± 43.9 months. Overall, 52.6% of participants were female, and 8.4% were smokers. The distribution of DR stages was as follows: Grade 1: 40.1%, Grade 2: 38.9%, and Grade 3: 21.0% (Table 1).

**TABLE 1:** Demographic characteristics, clinical parameters, and treatment distribution of the participants.

		**Count**	**Column N %**

Group	Control	151	49.0%
	DR	157	51.0%

Age (year), ±S.D.		61.6 ± 6.0	

BMI (kg/m^2^), ±S.D.		26.3 ± 4.4	

DM duration (month), ±S.D.		110.7 ± 43.9	
Gender	Female	162	52.6%
	Male	146	47.4%

Smoking	No	282	91.6%
	Yes	26	8.4%

DR stage	Grade 1	63	40.1%
	Grade 2	61	38.9%
	Grade 3	33	21.0%

Treatment	Int. Vit. Anti‐VEGF	270	87.7%
	Int. Vit. Anti‐VEGF + LP	38	12.3%

*Note:* Int. Vit.: intravitreal.

Compared to controls, the DR group had higher baseline FBG (126.0 ± 59.2 vs. 89.2 ± 5.0 mg/dL), LDL, TG, HbA1c, neutrophil, platelet counts, and macular thickness. Following treatment, significant improvements were observed in FBG (107.4 ± 54.5 ⟶ 107.8 ± 42.4 mg/dL), LDL, TG, HbA1c (7.0 ± 2.0 ⟶ 6.0 ± 1.4%), and macular thickness (376.5 ± 69.6 ⟶ 263.3 ± 59.3 μm) (all *p* < 0.05). Lymphocyte and monocyte counts also increased significantly. HDL levels showed no statistically significant change (*p* = 0.138) (Table 2).

**TABLE 2:** Intergroup comparison of biochemical, hematological, and retinal parameters before and after treatment.

	**Control (*n* = 151)**	**Pre-Tx (*n* = 157)**	**1 Month Tx (*n* = 157)**	**3 Month Tx (*n* = 157)**	**p** **value**

FBG (mg/dL)	89.2 ± 5.0	126.0 ± 59.2	107.4 ± 54.5	107.8 ± 42.4	**< 0.001**
LDL (mg/dL)	98.4 ± 16.9	114.8 ± 30.1	104.8 ± 23.9	103.4 ± 20.5	**< 0.001**
HDL (mg/dL)	56.1 ± 6.8	52.7 ± 11.1	54.6 ± 8.9	56.3 ± 9.1	0.138
Total Chol. (mg/dL)	174.3 ± 14.5	195.2 ± 34.5	179.3 ± 21.0	178.4 ± 18.1	**0.036**
TG (mg/dL)	110.9 ± 22.9	144.6 ± 51.7	118.0 ± 48.3	115.1 ± 29.6	**0.017**
HbA1c (%)	5.2 ± 0.2	7.0 ± 2.0	6.0 ± 1.5	6.0 ± 1.4	**< 0.001**
Neutrophil (× 10^3^/μL)	3.74 ± 1.3	6.01 ± 1.7	6.94 ± 2.1	5.96 ± 2.3	0.060
Lymphocyte (× 10^3^/μL)	2.48 ± 0.6	2.22 ± 0.5	2.43 ± 0.7	2.80 ± 0.7	**< 0.001**
Monocyte (× 10^3^/μL)	0.54 ± 0.2	0.52 ± 0.2	0.72 ± 0.5	0.75 ± 0.3	**< 0.001**
Platelet (× 10^3^/μL)	275.7 ± 80.7	289.8 ± 58.2	309.7 ± 118.9	335.9 ± 103.2	**< 0.001**
Macular thickness (μm)	254.2 ± 15.7	376.5 ± 69.6	308.4 ± 63.1	263.3 ± 59.3	**< 0.001**

*Note:* Post hoc analysis results (Bonferroni‐adjusted): FBG (mg/dL): control vs Pre‐Tx *p* < 0.001, Pre‐Tx vs 1 month Tx *p* = 0.002, and Pre‐Tx vs 3 month Tx *p* = 0.004. LDL (mg/dL): control vs Pre‐Tx *p* < 0.001, Pre‐Tx vs 1 month Tx *p* = 0.006, and Pre‐Tx vs 3 month Tx *p* = 0.004. Total cholesterol (mg/dL): control vs Pre‐Tx *p* = 0.015, Pre Tx vs 1 month Tx *p* = 0.022, and Pre‐Tx vs 3 month Tx *p* = 0.018. TG (mg/dL): control vs Pre‐Tx *p* < 0.001, Pre‐Tx vs 1 month Tx *p* = 0.011, and Pre‐Tx vs 3 month Tx *p* = 0.006. HbA1c (%): control vs Pre‐Tx *p* < 0.001, Pre‐Tx vs 1 month Tx *p* < 0.001, and Pre‐Tx vs 3 month Tx *p* < 0.001. Lymphocyte (× 10^3^/μL): control vs Pre‐Tx *p* = 0.004, Pre‐Tx vs 1 month Tx *p* = 0.041, Pre‐Tx vs 3 month Tx *p* < 0.001, and 1 month Tx vs 3 month Tx *p* = 0.038. Monocyte (× 10^3^/μL): Pre‐Tx vs 1 month Tx *p* = 0.013, Pre‐Tx vs 3 month Tx *p* = 0.008, control vs 1 month Tx *p* = 0.021, and control vs 3 month Tx *p* = 0.015. Platelet (× 10^3^/μL): Pre‐Tx vs 1 month Tx *p* = 0.047, Pre‐Tx vs 3 month Tx *p* < 0.001, control vs 3 month Tx *p* = 0.009, and 1 month Tx vs 3 month Tx *p* = 0.032. Macular thickness (μm): control vs Pre‐Tx *p* < 0.001, Pre‐Tx vs 1 month Tx *p* < 0.001, Pre‐Tx vs 3 month Tx *p* < 0.001, and 1 month Tx vs 3 month Tx *p* = 0.012. Bold values indicate statistically significant results (*p* < 0.05).

When compared with the control group, patients with DR demonstrated significantly higher baseline levels of systemic inflammatory indices, including NLR, MLR, PLR, and SII. After treatment, all indices showed a declining trend. NLR decreased from 3.1 ± 1.7 to 2.2 ± 1.0 (*p* = 0.028), MLR increased transiently but returned to near‐baseline by month 3 (*p* < 0.001), PLR declined from 144.3 ± 67.3 to 121.3 ± 29.8 (*p* < 0.001), and SII decreased significantly from 964.5 ± 761.0 to 736.2 ± 386.0 (*p* = 0.005) (Table 3).

**TABLE 3:** Temporal changes in systemic inflammatory indices before and after treatment.

	**Control (*n* = 151)**	**Pre-Tx (*n* = 157)**	**1 Month Tx (*n* = 157)**	**3 Month Tx (*n* = 157)**	**p** **value**

NLR	1.5 ± 0.3	3.1 ± 1.7	3.0 ± 1.0	2.2 ± 1.0	**0.028**
MLR	0.22 ± 0.04	0.27 ± 0.2	0.31 ± 0.26	0.27 ± 0.1	**<** **0.001**
PLR	112.0 ± 19.2	144.3 ± 67.3	129.8 ± 45.9	121.3 ± 29.8	**<** **0.001**
SII	416.4 ± 153.9	964.5 ± 761.0	895.9 ± 274.6	736.2 ± 386.0	**0.005**

*Note:* Data are presented as mean ± SD. Post hoc analysis results (Bonferroni‐adjusted): NLR: control vs Pre‐Tx *p* < 0.001 and Pre‐Tx vs 3 month Tx *p* = 0.019. MLR: Pre‐Tx vs 1 month Tx *p* = 0.007 and Pre‐Tx vs 3 month Tx *p* = 0.011. PLR: control vs Pre‐Tx *p* = 0.003, Pre‐Tx vs 1 month Tx *p* = 0.014, and Pre‐Tx vs 3 month Tx *p* < 0.001. SII: control vs Pre‐Tx *p* < 0.001 and Pre‐Tx vs 3 month Tx *p* = 0.005. Bold values indicate statistically significant results (*p* < 0.05).

Abbreviations: NLR, neutrophil‐to‐lymphocyte ratio; MLR, monocyte‐to‐lymphocyte ratio; PLR, platelet‐to‐lymphocyte ratio; SII, systemic immune‐inflammation index.

Pretreatment NLR showed a weak but statistically significant correlation with HbA1c (*r* = 0.150, *p* = 0.008) and a strong correlation with macular thickness (*r* = 0.826, *p* < 0.001). MLR, PLR, and SII did not correlate significantly with HbA1c but showed moderate to strong correlations with macular thickness (*p* < 0.001 for all) (Table 4).

**TABLE 4:** Correlation of pretreatment systemic inflammatory markers with HbA1c and macular thickness.

		**HbA1c (%)**	**Macular thickness (μm)**

Pre‐Tx NLR	Pearson Correlation	0.150	0.826
	*p* value	**0.008**	**< 0.001**

Pre‐Tx MLR	Pearson Correlation	−0.016	0.602
	*p* value	0.779	**< 0.001**

Pre‐Tx PLR	Pearson Correlation	0.018	0.694
	*p* value	0.747	**< 0.001**

Pre‐Tx SII	Pearson Correlation	0.090	0.776
	*p* value	0.116	**< 0.001**

*Note:* Bold values indicate statistically significant results (*p* < 0.05).

At the third month, no systemic inflammatory markers showed significant correlation with HbA1c (all *p* > 0.05). However, NLR (*r* = 0.172, *p* = 0.037), PLR (*r* = 0.179, *p* = 0.030), and SII (*r* = 0.265, *p* = 0.001) demonstrated weak but statistically significant correlations with macular thickness. MLR showed no correlation with either parameter (Table 5).

**TABLE 5:** Correlation of systemic inflammatory markers with HbA1c and macular thickness at the third month posttreatment.

		**HbA1c (%)**	**Macular thickness (μm)**

3‐Month Post‐Tx NLR	Pearson Correl.	−0.039	0.172
	*p* value	0.641	**0.037**

3‐Month Post‐Tx MLR	Pearson Correl.	−0.016	−0.004
	*p* value	0.847	0.961

3‐Month Post‐Tx PLR	Pearson Correl.	−0.082	0.179
	*p* value	0.319	**0.030**

3‐Month Post‐Tx SII	Pearson Correl.	−0.096	0.265
	*p* value	0.247	**0.001**

*Note:* Bold values indicate statistically significant results (*p* < 0.05).

**FIGURE 1:**
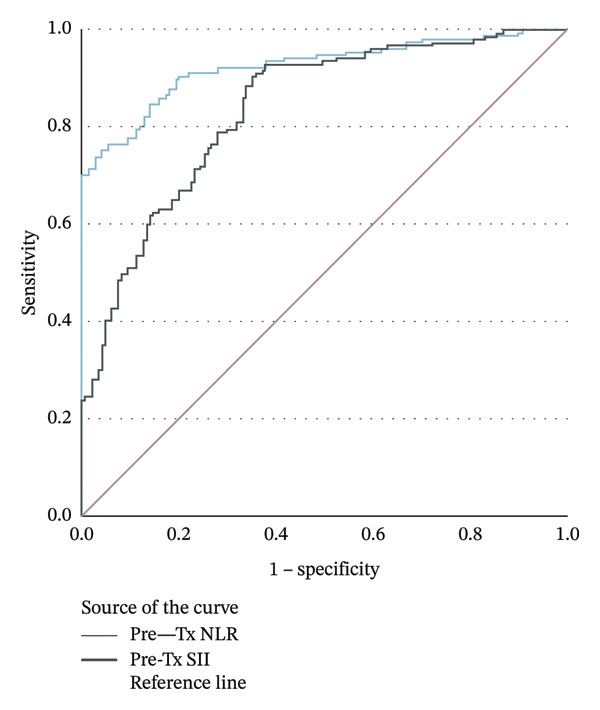
Receiver operating characteristic (ROC) curves of pretreatment neutrophil‐to‐lymphocyte ratio (pre‐TX NLR) and pretreatment systemic immune‐inflammation index (pre‐TX SII) for the prediction of the study outcome. The diagonal line represents the reference line.

ROC analysis revealed that pretreatment NLR had excellent diagnostic performance for DR (AUC = 0.925, sensitivity = 86%, specificity = 84%, and cut‐off = 1.82, *p* < 0.001). SII also showed good diagnostic accuracy (AUC = 0.838, sensitivity = 78%, specificity = 73%,and cut‐off = 495.7, *p* < 0.001) (Table 6, Figure 1).

**TABLE 6:** Diagnostic performance of pretreatment inflammatory markers in diabetic retinopathy based on ROC curve analysis.

	**Area**	**Sensitivity**	**Specificity**	**Cutoff**	**p** **value**	**Asymptotic 95% CI**	
						**Lower bound**	**Upper bound**

Pre‐Tx NLR	0.925	%86	%84	1.82	**<** **0.001**	0.895	0.955
Pre‐Tx SII	0.838	%78	%73	495.7	**<** **0.001**	0.794	0.882

*Note:* Bold values indicate statistically significant results (*p* < 0.05).

In multivariate analysis, pretreatment NLR (*β* = 1.201, *p* < 0.001), MLR (*β* = −0.418, *p* < 0.001), DM duration (*β* = 0.059, *p* = 0.048), and HbA1c (*β* = 0.099, *p* = 0.002) were identified as significant independent predictors of baseline macular thickness. Age, BMI, PLR, and SII showed no significant association (*p* > 0.05 for all) (Table 7).

**TABLE 7:** Multivariate linear regression analysis for predicting pretreatment macular thickness.

	**B**	**Std. error**	**Beta**	**t**	**p** **value**

Age (year)	0.451	0.39	0.034	1.157	0.248
BMI (kg/m^2^)	−0.288	0.545	−0.016	−0.528	0.598
DM duration (month)	0.106	0.058	0.059	1.83	**0.048**
Pre‐Tx NLR	66.083	7.248	1.201	9.118	**< 0.001**
Pre‐Tx MLR	−268.168	47.059	−0.418	−5.699	**< 0.001**
Pre‐Tx PLR	0.129	0.16	0.085	0.81	0.419
Pre‐Tx SII	−0.016	0.02	−0.123	−0.781	0.435
Pre‐Tx HbA1c (%)	4.707	1.534	0.099	3.068	**0.002**

*Note:* Bold values indicate statistically significant results (*p* < 0.05).

## 4. Discussion

This study investigated the relationship between systemic inflammatory biomarkers and structural changes in the retina among patients with newly diagnosed DR. In the pretreatment period, patients exhibited poor glycemic control, and inflammation‐related parameters (NLR, MLR, PLR, and SII) were found to be significantly elevated. Throughout the course of treatment, improvements were observed in both biochemical and hematological parameters, accompanied by a significant reduction in macular thickness. The positive correlation between NLR and HbA1c levels with macular thickness in the pretreatment phase supports the potential role of systemic inflammation in the development of retinal edema. Moreover, in multivariate regression analysis, NLR, HbA1c, and MLR were found to have independent effects on macular thickness, reinforcing the utility of these biomarkers in predicting structural damage. Our findings suggest that systemic inflammation plays a significant role in the early phase of DR, not only in diagnosis but also in determining disease severity.

In addition, the pathogenesis of DR is intricate and driven by multiple interrelated mechanisms, including prolonged hyperglycemia, oxidative stress, and persistent inflammation. Among these, chronic low‐grade inflammation plays a pivotal role, contributing significantly to the breakdown of the blood–retinal barrier, endothelial cell impairment, the upregulation of adhesion molecules, and enhanced infiltration of inflammatory cells into the retinal tissue [14, 15]. Elevated inflammatory activity and immune responses promote the expression of various pro‐inflammatory mediators and chemokines, notably interleukin‐1β (IL‐1β), tumor necrosis factor‐alpha (TNF‐α), and vascular endothelial growth factor (VEGF). These factors further aggravate endothelial dysfunction, facilitate leukocyte migration, and drive pathological retinal neovascularization associated with DR [16, 17]. Platelets are actively involved in the inflammatory cascade by secreting pro‐inflammatory mediators and promoting endothelial impairment. An elevated PLR has been linked to both the occurrence and progression of DR, indicating an enhanced systemic inflammatory response and a greater susceptibility to microvascular complications in individuals with DM [18, 19].

Furthermore, in the study conducted by Dascalu et al. (2023), NLR values were shown to be significantly associated with the presence and severity of DR [20]. A marked increase in NLR levels was particularly observed in the proliferative stage, while PLR demonstrated borderline significance. These findings are consistent with our study, in which both NLR and PLR levels were significantly elevated during the pretreatment period. Furthermore, in our study, the positive correlation between NLR and macular thickness, as well as the identification of NLR as an independent predictor of macular edema in multivariate analysis, supports the prognostic role of NLR emphasized by Dascalu and colleagues. However, the fact that PLR was not as strong a determinant in either study suggests that its diagnostic value may be more limited. The shared conclusion that NLR may reflect systemic inflammation and influence the progression of retinopathy highlights the central role of inflammatory processes in the pathogenesis of DR, as demonstrated by both studies. These mechanisms are consistent with the current understanding of DR classification and pathophysiology outlined in recent consensus literature [11].

Similarly, neutrophil‐to‐lymphocyte ratio and PLR are two parameters recognized as novel biomarkers of systemic inflammatory response, based on the distribution of different leukocyte subpopulations in peripheral blood [21]. Multiple studies have demonstrated that these hematological markers are associated with type 2 DM (T2DM) [22, 23]. Moreover, existing evidence indicates that NLR and PLR are significantly associated with diabetes and its related complications and serve as practical, easily applicable biomarkers [22, 24]. Some studies have shown that PLR is closely related to macrovascular and microvascular complications observed in advanced stages of diabetes [25–27]. However, conflicting results also exist; for instance, one study found no significant association between PLR and DR [25]. In a study by Atlı et al., NLR and PLR levels were found to be significantly elevated in the proliferative DR group [28]. This finding aligns with our results, where both NLR and PLR were significantly higher in the pretreatment period compared to the control group. Moreover, Atlı and colleagues suggested that inflammation plays a central role in the pathogenesis of DR and that these parameters may serve as indicators of disease progression. Our findings further support this view, as NLR was not only elevated prior to treatment but also showed a positive correlation with macular thickness and emerged as a significant predictor in the regression model. The reduction in systemic inflammatory indices observed after intravitreal anti‐VEGF therapy is more likely attributable to the resolution of retinal inflammation—an upstream driver of systemic cytokine release—rather than to a direct systemic anti‐inflammatory effect of the drug [29]. This interpretation is consistent with previous studies demonstrating the limited systemic bioavailability of intravitreally administered anti‐VEGF agents [30]. These pharmacologic considerations align with contemporary DR management pathways summarized in recent consensus statements [11]. Although this remains to be clarified in future studies, it highlights the potential systemic impact of localized ocular interventions.

Accumulating evidence suggests that systemic inflammation is a key contributor to the pathogenesis and advancement of DR. Higher NLR values have been associated with suboptimal glycemic regulation and an increased inflammatory state in individuals with diabetes, underscoring its role as a marker of systemic inflammation [31]. Moreover, serum inflammatory biomarkers have been correlated with the severity of retinopathy, suggesting that chronic low‐grade inflammation contributes to retinal microvascular damage [32]. In accordance with the existing evidence, anti‐inflammatory agents, such as nonsteroidal anti‐inflammatory drugs, have been reported to mitigate retinal inflammatory responses and decelerate disease progression in experimental models and clinical studies [33]. These findings are consistent with our results showing that higher systemic inflammatory indices are linked with greater macular thickness, while their reduction following treatment corresponds with retinal improvement.

Several limitations should be acknowledged, including the single‐center nature of the study and the modest sample size, which may restrict the broader applicability of the findings. Inflammatory markers were assessed solely through peripheral blood samples, which may not fully reflect the impact of these parameters on local retinal inflammation. The follow‐up period was restricted to 3 months, limiting the ability to evaluate the long‐term effects of inflammatory parameters. In addition, some potential confounding variables that may influence inflammation levels—such as subclinical infections; microvascular complications of diabetes; or variations in medication type, dosage, and duration (including antihyperglycemic, antihypertensive, and lipid‐lowering agents)—may not have been fully controlled. Considering all these limitations, there is a clear need for longer‐term, multicenter prospective studies to validate and expand upon these findings.

This study demonstrated the temporal changes in systemic inflammatory indices and their relationship with retinal structural alterations in newly diagnosed DR patients. The findings revealed that markers such as NLR, MLR, and HbA1c were significantly associated with macular thickness and were elevated during the pretreatment period. Following 3 months of anti‐VEGF therapy, substantial improvements were observed in both systemic inflammation levels and retinal thickness. In addition, pretreatment NLR and SII values showed high sensitivity and specificity in differentiating DR, indicating their diagnostic potential. The results suggest that systemic inflammation may serve as a valuable biomarker not only in the pathophysiology of DR but also in monitoring disease progression and treatment response. In this context, easily accessible and cost‐effective parameters like NLR may support personalized patient management if integrated more widely into clinical practice. Further large‐scale, multicenter prospective studies with extended follow‐up periods are warranted to validate and expand upon these findings.

From a clinical standpoint, monitoring systemic inflammatory indices alongside retinal imaging may offer a more comprehensive approach to patient evaluation and follow‐up. Integrating such hematologic markers into clinical protocols could enable earlier identification of high‐risk patients and more individualized treatment planning.

## Author Contributions

Emre Aydın, Şaban Kılıç, and Çiğdem Deniz Genç contributed to the conception and design of the study. Emre Aydın and Şaban Kılıç were responsible for data collection and patient follow‐up. Emre Aydın and Çiğdem Deniz Genç performed statistical analysis and interpretation. Emre Aydın drafted the manuscript.

## Funding

This research received no specific grant from any funding agency in the public, commercial, or not‐for‐profit sectors.

## Disclosure

All authors reviewed and approved the final version of the manuscript.

## Conflicts of Interest

The authors declare no conflicts of interest.

## Data Availability

The data that support the findings of this study are available from the corresponding author upon reasonable request.
