# Correction to “Monitoring Systemic Inflammatory Indices and Retinal Changes Over Time Following Initial Diagnosis of Diabetic Retinopathy”

**DOI:** 10.1155/joph/9848609

**Published:** 2026-06-12

E. Aydın, Ş. Kılıç, and Ç. D. Genç, “Monitoring Systemic Inflammatory Indices and Retinal Changes Over Time Following Initial Diagnosis of Diabetic Retinopathy,” *Journal of Ophthalmology* 2026 (2026): 5542064, https://doi.org/10.1155/joph/5542064.

In the abovementioned article, there was an error in affiliation 1, where the institution was listed as “Samsun Education and Research Hospital.” The correct institution is “Samsun University.” The correct version of affiliation 1 is shown below:

Department of Ophthalmology, Samsun University, Samsun, Turkey

This has been updated on the published article.

We apologize for this error.

